# Associations Between Cardiorespiratory Fitness and Metabolic Syndrome in Adolescents: A Systematic Review and Meta-Analysis

**DOI:** 10.3390/metabo14110635

**Published:** 2024-11-18

**Authors:** Jonathan Cesar do Prado, Dartagnan Pinto Guedes, Pedro Henrique Garcia Dias, Antonio Stabelini Neto, Raphael Gonçalves de Oliveira

**Affiliations:** 1 Postgraduate Program in Physical Exercise in Health Promotion, Universidade Norte do Paraná (UNOPAR), Londrina 86041-120, PR, Brazil; jonathanprado2991@gmail.com; 2 Postgraduate Program in Human Movement Sciences, Universidade Estadual do Norte do Paraná (UENP), Jacarezinho 86400-000, PR, Brazil; dartagnan.guedes@uenp.edu.br (D.P.G.); ph.garciadias@gmail.com (P.H.G.D.); asneto@uenp.edu.br (A.S.N.)

**Keywords:** metabolic risk, cardiovascular diseases, physical fitness, young population

## Abstract

**Introduction:** Low levels of cardiorespiratory fitness (CRF) are associated with a greater risk of metabolic syndrome (MetS) in adolescence. In this sense, it is important to verify the strength of this association and the certainty that this evidence can be recommended. **Objective:** The objective of this paper is to summarize, through a systematic review and meta-analysis, the evidence available to verify the association between CRF and MetS in adolescents. **Methods:** PubMed, Embase, CINAHL, SPORTDiscus, LILACS, and Web of Science were searched until 20 August 2024. The risk of bias in each study was assessed via the AXIS tool, and the certainty of the evidence was assessed via the GRADE system. For the meta-analysis, the odds ratio (OR) was calculated with a 95% confidence interval. **Results:** Nine studies (7077 participants), all with a low risk of bias, were included in the systematic review. There was a high certainty of evidence that adolescents with low CRF have significantly greater odds of developing MetS (OR = 3.63 [CI 95%, 2.54 to 5.20]). The odds increase for low vs. moderate (OR = 4.23 [CI 95%, 2.64 to 6.78]) and low vs. high (OR = 8.03 [CI 95%, 3.20 to 20.18]) CRF are considered separately. The effect does not change according to the type of test used to assess CRF (*p* = 0.51). **Conclusions:** There is high certainty of evidence that adolescents with low CRF levels have significantly greater odds of developing MetS; therefore, it is essential that intervention strategies be designed to increase CRF in this population.

## 1. Introduction

Metabolic syndrome (MetS) is characterized by a set of interrelated risk factors, including abdominal obesity, hypertension, elevated triglycerides and fasting glucose, and reduced HDL cholesterol. The presence of three of these factors defines the presence of MetS, which can double the risk of exposure to cardiovascular diseases and increase the risk of type II diabetes mellitus fivefold in the 5–10 years after diagnosis [1]. Considering that cardiovascular diseases are the leading cause of death worldwide, early diagnosis of the possible presence of MetS in adolescence is essential, which allows for the design and implementation of intervention strategies that can reduce the mortality rate from cardiovascular diseases in adulthood [2].

Among the factors capable of preventing MetS, the maintenance of adequate levels of cardiorespiratory fitness (CRF) [3] stands out as an important marker of health in young people and adults [4]. CRF reflects the integrated capacity of the body to transport oxygen from the atmosphere to the muscle and use it as an energy source in prolonged muscular activity, typically translated as the maximum oxygen volume (VO_2max_). Although it is widely recognized as an important marker of cardiovascular risk, it is the only important risk factor not routinely assessed in clinical practice [5].

A previous longitudinal study revealed that individuals with persistently low CRF or who decreased their CRF between adolescence (15 years) and adulthood (33 years) were 11.5 and 8.3 times more likely to develop MetS, respectively [6]. Furthermore, as demonstrated by a previously published meta-analysis of observational evidence, low CRF at a young age appears to be a determining factor for the presence of MetS in adolescence [7]. At the time, only five studies were available for meta-analysis, and the certainty of the evidence was not assessed [8,9,10,11,12]. Since then, observational evidence has consistently confirmed that low CRF levels in adolescence pose a greater risk for MetS [6,13,14,15].

Therefore, it is necessary to update the grouping of the effect sizes of the different studies to indicate to what extent a low level of CRF is associated with greater chances of identifying MetS. In addition, it is essential to clarify the certainty of this evidence around the grouped effect among the different studies. Therefore, the objective of the present study was to verify the association between CRF and MetS in adolescents and to identify the level of recommendation around the observed effect.

## 2. Methods

This systematic review and meta-analysis was written according to the recommendations of the PRISMA protocol [16] and was prospectively registered in PROSPERO (CRD42024570653). The inclusion criteria were as follows: (a) observational studies (cross-sectional, case–control, and prospective); (b) adolescent participants (aged 10–19 years); (c) studies that identified MetS while accounting for criteria adapted for adolescents; and (d) studies that associated MetS with a valid measure of CRF.

The exclusion criteria were as follows: (a) studies with data already available in another included study (identical data from the same population published two or more times); (b) studies that did not diagnose MetS or that considered only a continuous risk score; (c) studies in which MetS was not associated with the variable of interest (CRF); and (d) studies that included adolescents with musculoskeletal or neurodegenerative diseases or physical or mental disabilities.

### 2.1. Databases and Search Strategy

The databases searched were PubMed, Embase, CINAHL, SPORTDiscus, LILACS, and Web of Science, without filtering for publication date and language. The search was last updated on 20 August 2024. The following descriptors were used for searching: (“metabolic syndrome” OR “metabolic syndrome x” OR “syndrome x”) AND (“cardiorespiratory fitness” OR “cardiorespiratory endurance” OR “cardiorespiratory test” OR “aerobic fitness”) AND (“adolescent” OR “youth” OR “teen” OR “teenager”). The complete search strategy used in each database can be accessed in the Supplementary Material. The references of the included studies were checked in an attempt to locate additional reports.

### 2.2. Study Selection

One reviewer performed the initial search in the databases (RGO), extracting titles and abstracts. A second reviewer (JCP) subsequently excluded duplicates via reference software (https://www.rayyan.ai/). Two reviewers (JCP and PHGD) subsequently blindly read the titles and abstracts to verify potential reports that met the inclusion criteria. The reports that passed the previous stage were subsequently read in full, blindly, by the same reviewers to define which ones would be included in the systematic review. In all stages, disagreements were resolved by consensus, or when there was no consensus, they were transferred to a third reviewer (RGO).

### 2.3. Data Extraction

The following information was extracted blindly by two reviewers (JCP and PHGD): (a) study identification (author, year, and country); (b) participants (sample size, age, and sex); (c) observational study design (case–control, cross-sectional, or prospective); (d) criteria for the diagnosis and prevalence of MetS; (e) method of CRF assessment; (f) variables used statistically for adjustment; and (g) main results. Discrepancies in the extraction of these data were resolved by consensus among the reviewers or by transfer to a third party (RGO).

### 2.4. Assessment of the Risk of Bias in the Included Studies

The AXIS (Appraisal Tool for Cross-Sectional Studies) tool [17] was used to assess the risk of bias in the included studies. This tool consists of 20 components that allow for critical evaluation of different elements of a published report to identify the lack of information that may increase the risk of bias. Two reviewers (JCP and PHGD) evaluated each item blindly, with disagreements being resolved by consensus. On the basis of the final score obtained, the studies were classified as high methodological quality (16–20 points), moderate (11–15 points), low (6–10 points), or very low (0–5 points).

### 2.5. Statistical Analysis

For the meta-analysis, the effect measure was calculated as the odds ratio (OR), with a 95% confidence interval (95% CI). The Cochrane Q test was performed to verify heterogeneity, with a statistical significance of *p* ≤ 0.10. Heterogeneity was also quantified via the I^2^ statistic, where ≤40% may not be important, 30–60% may indicate moderate heterogeneity, 50–90% may represent high heterogeneity, and ≥75% indicates considerable heterogeneity [18]. When no statistically significant heterogeneity was identified, a fixed effects model was used; otherwise, a random effects model was used. Statistical significance for the effect size of the association was considered at *p* < 0.05. It was not possible to assess possible publication bias via the funnel plot, since at least 10 studies were not included in the meta-analysis [18,19]. All analyses were performed via Review Manager (RevMan) [Computer program], version 5.3, Copenhagen: The Nordic Cochrane Centre, The Cochrane Collaboration.

### 2.6. Analysis of the Certainty of Evidence

The certainty of the evidence was graded according to the Grading of Recommendations, Assessment, Development, and Evaluation (GRADE) [19]. Two independent reviewers (RGO and JCP) performed the analysis blindly, with disagreements being resolved later by consensus. GRADE has domains that allow for decreasing or increasing the certainty of the evidence, considering (a) limitations in the design or execution of the study (risk of bias); (b) inconsistency in the results; (c) indirectness; (d) imprecision; and (e) other factors (publication bias, dose–response gradient, magnitude of effect, and confounding factors).

In this way, it is possible to generate a final classification of certainty around the observed evidence, which can be (a) high (future research is unlikely to change the estimate or confidence in the results); (b) moderate (new research will likely have an impact on confidence in the estimate of the effect and may even modify the estimate); (c) low (future research will likely have a significant effect on confidence in the estimate of the effect and will change the estimate); or (d) very low (the results are highly uncertain).

### 2.7. Sensitivity and Subgroup Analyses

Sensitivity analysis was performed to verify the possible influence of studies that did not adjust for confounding factors. Furthermore, sensitivity analysis was planned to verify the possible influence of studies with low methodological quality (high risk of bias). However, since no study obtained a score ≤ 10 points using the AXIS tool, this analysis was not necessary. We also performed sensitivity analysis by excluding studies one by one, in order to verify whether any specific study could be significantly affecting the results. In addition, we performed sensitivity analysis by excluding studies with greater weight in the meta-analysis, in order to confirm the robustness of the findings.

Subgroup analyses were conducted in two situations: (1) to separately analyze studies that compared moderate vs. low CRF and high vs. low CRF and (2) to separately analyze studies by the type of test used to measure CRF (a—maximal treadmill or cycle ergometer test; b—maximal field test [20 m shuttle run]; c—submaximal treadmill or cycle ergometer test).

## 3. Results

**Figure 1:**
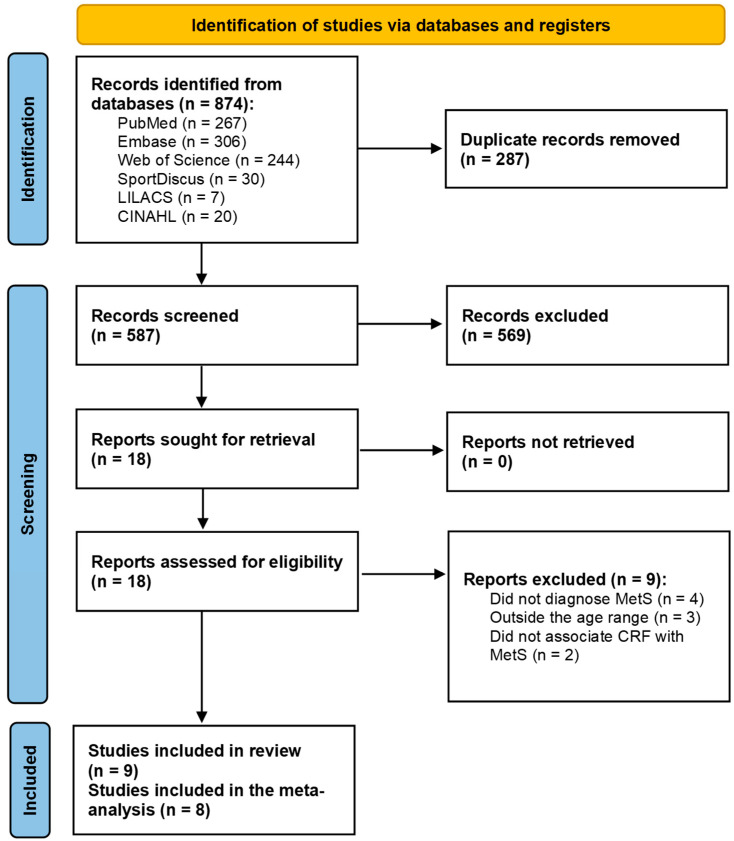
Prism diagram.

Initially, 874 reports were identified in the databases. After the exclusion of duplicates, 587 reports remained after the titles and abstracts were read, of which 569 were excluded. All 18 remaining full texts were located and read in full. After reading, nine studies did not meet the inclusion criteria. Thus, nine studies (each represented by a report) were included in this systematic review, and eight were included in the meta-analysis (Figure 1). No additional reports were located after the references of the included studies were read.

Table 1 presents a summary of the included studies. The year of publication ranged from 2007 to 2021, and the studies were conducted in Europe [6,10,12,13,15], North America [8,9], South America [11], and Asia [14]. The total number of participants was 7077, ranging from 44 to 2446 across studies. Most studies were cross-sectional, with the exception of two prospective studies [6,9]. MetS was diagnosed mainly by harmonized criteria [6,12,14] and the International Diabetes Federation (IDF) [10,13,15], while the prevalence rate ranged from 0.8% to 27.3% across the nine studies. CRF was assessed mainly by the PACER/20 m shuttle run test [11,13,14,15], in addition to maximal [6,10,12] and submaximal [8,9] tests on a treadmill or cycle ergometer. For the analyses of the associations between CRF and MetS, only two studies did not adjust for possible confounding variables [12,13]. All studies revealed a significant association between adolescents with low CRF and increased odds of MetS or moderate/high CRF as a protective effect against MetS. Regarding the risk of bias in the studies, there was a variation between 11 and 18 points.

### Quantitative Synthesis (Meta-Analysis)

**Figure 2:**
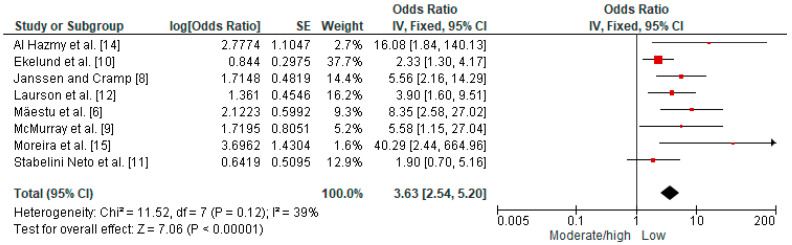
Forest plot of the primary analysis demonstrating the odds ratio for metabolic syndrome among adolescents with moderate/high vs. low cardiorespiratory fitness.

The primary analysis demonstrated a significant association, with high certainty of evidence (Table 2), that low CRF increases the chance of adolescents having MetS by more than three times (OR = 3.63 [CI 95%, 2.54 to 5.20] *p* < 0.00001, n = 6868, studies = 8, I^2^ = 39%; Figure 2). Subgroup analysis, which separately verified moderate vs. low CRF (OR = 4.23 [CI 95%, 2.64 to 6.78] *p* < 0.00001, n = 3861, studies = 5, I^2^ = 5%) and high vs. low CRF (OR = 8.03 [CI 95%, 3.20 to 20.18] *p* < 0.00001, n = 3861, studies = 5, I^2^ = 65%), demonstrated that the chance of adolescents with low CRF having MetS may be four and eight times greater, respectively (Supplementary Figure S1). Subgroup analysis, which sought to separately verify the studies by the type of test used to measure CRF, demonstrated that the significant association with MetS remained, regardless of the test used, with no significant difference (*p* = 0.51) between the forms of application (Supplementary Figure S2). For the sensitivity analysis that removed one study [12] that did not adjust for possible confounding variables, no change in results was observed. The results also did not change for the other sensitivity analyses when excluding each study one by one and removing the studies with the greatest weight in the meta-analysis.

## 4. Discussion

The aim of the present study was to verify the association between CRF and MetS in adolescents and to identify the level of recommendation around the observed effect. In summary, our findings demonstrated a high certainty of evidence that adolescents with low CRF may have a three- to four-fold greater chance of being exposed to MetS. Notably, our subgroup analyses revealed a dose–response gradient, in which the chance of adolescents with low CRF being diagnosed with MetS was eight times greater than that of their peers with high CRF.

Our findings are in line with a previous meta-analysis, which included five observational studies in the meta-analysis and, at the time, identified four times greater chances of adolescents with low CRF presenting with MetS [7]. This update advances by analyzing the certainty of evidence via the GRADE system. In this case, a high certainty of evidence was identified, indicating that there is strong confidence that the true effect is close to the estimated effect. This implies that additional studies are unlikely to change the confidence in the effect estimate. Furthermore, unlike the previously published meta-analysis, the current study performed a subgroup analysis and indicated an important dose–response gradient, indicating that adolescents with high CRF may be even more protected from MetS than those classified as having moderate CRF.

This magnitude of effect was evident in the qualitative analysis of each study included in the current review, which classified adolescents into more than two levels of CRF (e.g., low, moderate, and high) [6,8,9,11,12]. In all the selected studies, a significant increase in the chance of identifying MetS was observed among adolescents with low vs. high CRF compared with those with low vs. moderate CRF. Most studies have used the tertile distribution of the sample itself to categorize adolescents into three different CRF levels, which makes it difficult to indicate a specific cutoff point that can be used in professional practice, for example, in the school environment.

One specific study used the FitnessGram test battery, which provides cutoff points according to the sex and age of adolescents, guiding the categorization of health zones. In this case, the healthy fitness zone can be used as a reference to be encouraged among adolescents [12]. The cutoff points for CRF equivalent to the health risk zone proposed by FitnessGram have good predictive power to identify adolescents at risk of MetS [23]. Among the tests that can be used, one of the best alternatives is the 20 m shuttle run, also known as PACER, as it is characterized as a field test that does not require sophisticated equipment other than a sound device that emits sound signals that determine the cadence/rhythm at each stage of the test progression. In addition, several adolescents can perform the test at the same time, which is particularly useful in a school environment. The test result can then be classified into the health zones proposed by the FitnessGram battery [24].

A prior classification allows for at least two immediate strategies: (1) refer adolescents classified as being in health risk zones, aiming to conduct more specific tests to confirm the diagnosis of MetS; (2) encourage adolescents classified as being in risk zones to increase their physical activity, especially of moderate–vigorous intensity, and consequently improve their CRF to change their classification in future evaluations. In addition, these adolescents should be encouraged to participate in physical exercise programs specifically designed and implemented for this purpose, since it has been demonstrated that combined physical exercise programs involving aerobic and resistance/muscle strengthening exercises performed twice a week for 12 weeks are capable of significantly improving CRF and other components of physical fitness in adolescents [25].

These actions aim to identify early those individuals at greatest risk for MetS, a cardiometabolic abnormality that affects 12.5% to 31.4% of the adult population, depending on the diagnostic criteria adopted. Thus, it is the gateway to the onset and progression of cardiovascular diseases and type II diabetes mellitus [26]. Cardiovascular diseases are the leading cause of death worldwide [27], whereas type II diabetes mellitus affects approximately 10% of the adult population and is responsible for substantially increasing the risk of vascular complications, ophthalmic abnormalities, and cardiovascular diseases [28]. Therefore, monitoring CRF in young adolescents may contribute as a viable alternative to reduce the incidence of these conditions in adulthood.

Finally, it should be considered that some adolescents may eventually have low CRF due to having MetS. In other words, they initially present characteristics such as insulin resistance and abdominal obesity that lead to low CRF. In this sense, interventions may also be necessary to help treat the conditions that are leading to low CRF, so that adolescents in this condition can reverse their current metabolic health status.

### Strengths and Limitations

A strong point of this study is the high certainty of the observed evidence, suggesting that future studies will probably not change the effect estimate. Another highlight is the scope of the search, which was conducted in six important databases without adding filters that could limit language or publication date. Furthermore, the subgroup analysis revealed an important dose–response relationship, which can provide support for professionals to encourage the improvement of CRF in adolescents with the aim of preventing MetS.

Regarding the limitations, it should be emphasized that there was no standardization of the diagnostic criteria adopted to identify MetS among the included studies, and there was no standardization for the cutoff points that classified adolescents in the different CRF strata. Finally, although most studies adjusted their analyses for potential confounding variables, only one study adjusted for measures of overweight/obesity, which could eventually change the magnitude of the observed effect.

## 5. Conclusions

Our findings demonstrated that there is high certainty in the evidence that adolescents with low CRF may be approximately four times more likely to be exposed to MetS. In this sense, it is essential to monitor this segment of the population through the routine application of tests designed to measure CRF to identify those at greatest risk so that they can then be referred for more specific evaluation and interventions if necessary.

## Figures and Tables

**Table 1:** Summary of studies included in the systematic review.

Study, Country	Sample (Number, Gender, Age)	Design	MetS (Criteria and Prevalence)	Cardiorespiratory Fitness Measure and Classification Criteria	Adjustments	Results (*p* < 0.05)	Risk of Bias 0–20
González-Gálvez et al. [13] (2021), Portugal	N = 209, 96 (M), 113 (F), 10–12 years	CS	IDF [20], MetS = 5.7%	VO_2Max_ (20 m shuttle run test): dichotomous classification (fit and unfit)	No adjustment	β −0.017 [SD 0.005] for lower CRF and MetS	16
Mäestu et al. [6] (2020), Estonia	N = 1076, 482 (M), 594 (F), 15 years	PRO (10 and 18 yrs)	Harmonized criteria * MetS = 2.2%	VO_2Peak_ (maximal treadmill test): quartile (high, high–moderate, low–moderate and low)	Length to follow-up, sex, and cohort	OR 34.43 [CI 95%, 4.62 to 256.85] and OR 11.52 [CI 95%, 3.79 to 34.98] for low (vs. high) CRF in adolescence (15 yrs) and MetS in adults (25 and 33 yrs, respectively)	17
Al Hazmy et al. [14] (2018), Indonesia	N = 44, 22 (M), 22 (F) 15–17 years	CS	Harmonized criteria * MetS = 27.3%	VO_2Max_ (20 m shuttle run test): dichotomous classification (fit and unfit)	Age	OR 16.08 [CI 95%, 1.84 to 140.13] for unfit CRF (vs. fit) and MetS	16
Laurson et al. [12] (2015), Hungary	N = 379, 213 (M), 166 (F), 12–18 years	CS	Harmonized criteria *, MetS = 6.7%	VO_2Peak_ (maximal treadmill test): FitnessGram classification (“Healthy Fitness Zone”; “Need to improve”; or “Needs Improvement/Risk Zone”)	No adjustment	OR 3.9 [CI 95%, 1.6–9.1] for “Needs Improvement” and MetS or OR 4.7 [CI 95%, 2.0 to 11.0] for “Needs Improvement/Risk Zone” and MetS	15
Stabelini Neto et al. [11] (2011), Brazil	N = 456, 233 (M), 223 (F), 10–18 years	CS	Cook [21], MetS = 7.7%	VO_2Max_ (20 m shuttle run test): tertiles (low, moderate, and high)	Age and sex	OR 3.0 [CI 95%, 1.13 to 7.94] for low CRF (vs. high) and MetS	18
Moreira et al. [15] (2010), Portugal	N = 517, 220 (M), 297 (F), 15–18 years	CS	IDF [20], MetS = 5%	PACER (20 m): FitnessGram classification (“below the healthy zone”; or “inside/above the healthy zone”)	Pubertal stage and socioeconomic status	OR 40.29 [CI 95%, 2.44 to 664.96] for CRF below the healthy zone (vs. inside/above the healthy zone) and MetS	16
Ekelund et al. [10] (2009), Denmark, Estonia, and Portugal	N = 2.446, NR (M), NR (F), 10–15 years	CS	IDF [20], MetS = 0.8%	Maximal cycle ergometer test: watts per fat-free mass, per minute	Age, sex, and nationality	OR 0.43 [CI 95%, 0.24 to 0.80] for high CRF and lower chance of MetS	15
McMurray et al. [9] (2008), USA	N = 389, 212 (M), 177 (F), 14–17 years (at follow-up)	PRO (7 yrs)	Jolliffe [22], MetS = 4.6% (at follow-up)	VO_2Max_ (submaximal test on cycle ergometer): absolute and/or tertile value (low, moderate, or high)	Sex, BMI, blood pressure, and cholesterol	OR 6.09 [CI 95%, 1.18 to 60.29] for low (vs. high) and OR 5.58 [CI 95%, 1.15 to 53.75] (vs. moderate) CRF in childhood (7–10 yrs) and MetS in adolescence (14–17 yrs)	16
Janssen and Cramp [8] (2007), USA	N = 1.561, 829 (M), 732 (F) 12–19 years	CS	Jolliffe [22], MetS = 7.6%	VO_2Max_ (submaximal treadmill test): tertiles (low, moderate, or high)	Age, sex, ethnicity, smoking, economic status, lipid and carbohydrate intake	OR 0.18 [CI 95%, 0.07 to 0.48] for moderate and OR 0.01 [CI 95%, 0.00 to 0.07] for high CRF (vs. low) and lower chance of MetS	11

M: male; F: female; NR: not reported; CS: cross-sectional study; PRO: prospective study; IDF: International Diabetes Federation; MetS: metabolic syndrome; CRF: cardiorespiratory fitness; VO_2Peak_: peak oxygen uptake; VO_2Max_: maximum oxygen consumption; PACER: progressive aerobic cardiovascular and endurance run; BMI: body mass index; OR: odds ratio; CI 95%: confidence interval of 95%; * harmonized criteria refers to the use of a combination of two or more criteria.

**Table 2:** Analysis of the certainty of evidence for the association between cardiorespiratory fitness and metabolic syndrome in adolescents.

Certainty Assessment						№ of Participants	OR (95% CI)	GRADE Certainty
№ of Studies	Risk of Bias	Inconsistency	Indirectness	Imprecision	Other Considerations			
8	not serious	not serious	not serious	not serious	strong association; plausible residual confounding would reduce the effect; dose response gradient	6868	3.63 (2.54, 5.20)	⨁⨁⨁⨁ High

OR: odds ratio; CI: confidence interval.

## Supplementary Materials

The following supporting information can be downloaded at https://www.mdpi.com/article/10.3390/metabo14110635/s1. Figure S1: Forest plot of subgroup analysis demonstrating the odds ratio for metabolic syndrome among adolescents with: (a) moderate vs low cardiorespiratory fitness; (b) high vs. low cardiorespiratory fitness. Figure S2: Forest plot of the subgroup analysis demonstrating the odds ratio for metabolic syndrome among adolescents with moderate/high vs low cardiorespiratory fitness according to the type of test applied.
