# Supplementary material for: Associations Between Cardiorespiratory Fitness and Metabolic Syndrome in Adolescents: A Systematic Review and Meta-Analysis
Source: Metabolites. 2024 Nov 18;14(11):635. doi: 10.3390/metabo14110635 (PMC11596233; doi:10.3390/metabo14110635)
Supplement: Supplementary file 1 [file metabolites-14-00635-s001.zip › metabolites-3306805-supplementary.pdf]

Supplementary Material

Search strategy in each database

Note: To increase search sensitivity we left "all fields" in each search term.

PubMed search strategy (pubmed.gov, 1996 to 20, August 2024).
#1: metabolic syndrome [MeSH Terms]
#2: metabolic syndrome x
#3: syndrome x
#4: #1 OR #2 OR #3
#5: cardiorespiratory fitness [MeSH Terms]
#6: cardiorespiratory endurance
#7: cardiorespiratory test
#8: aerobic fitness
#9: #5 OR #6 OR #7 OR #8
#10: adolescent [MeSH Terms]
#11: youth
#12: teen
#13: teenager
#14: #10 OR #11 OR #12 OR #13
#15: #4 AND #9 AND #14

Embase (embase.com, 1947 to 20, August 2024)
#1: metabolic syndrome/exp
#2: metabolic syndrome x
#3: syndrome x/exp
#4: #1 OR #2 OR #3
#5: cardiorespiratory fitness/exp
#6: cardiorespiratory endurance/exp
#7: cardiorespiratory test
#8: aerobic fitness/exp
#9: #5 OR #6 OR #7 OR #8
#10: adolescent/exp
#11: youth/exp
#12: teen
#13: teenager/exp
#14: #10 OR #11 OR #12 OR #13
#15: #4 AND #9 AND #14

CINAHL (via EBSCOhost, 1937 to 20, August 2024)
S1: metabolic syndrome
S2: metabolic syndrome x
S3: syndrome x
S4: S1 OR S2 OR S3
S5: cardiorespiratory fitness
S6: cardiorespiratory endurance
S7: cardiorespiratory test
S8: aerobic fitness
S9: S5 OR S6 OR S7 OR S8
S10: adolescent
S11: youth

S12: teen
S13: teenager
S14: S10 OR S11 OR S12 OR S13
S15: S4 AND S9 AND S14

SPORTDiscus (via EBSCOhost, 1985 to 20, August 2024)
S1: metabolic syndrome
S2: metabolic syndrome x
S3: syndrome x
S4: S1 OR S2 OR S3
S5: cardiorespiratory fitness
S6: cardiorespiratory endurance
S7: cardiorespiratory test
S8: aerobic fitness
S9: S5 OR S6 OR S7 OR S8
S10: adolescent
S11: youth
S12: teen
S13: teenager
S14: S10 OR S11 OR S12 OR S13
S15: S4 AND S9 AND S14

LILACS (lilacs.bvsalud.org, 1986 to 20, August 2024)
((metabolic syndrome) OR (metabolic syndrome x) OR (syndrome x)) AND ((cardiorespiratory fitness) OR (cardiorespiratory endurance) OR (cardiorespiratory test) OR (aerobic fitness)) AND ((adolescent) OR (youth) OR (teen) OR (teenager))

Web of Science (webofscience.com, 1900 to 20, August 2024)
1: metabolic syndrome
2: metabolic syndrome x
3: syndrome x
4: 1 OR 2 OR 3
5: cardiorespiratory fitness
6: cardiorespiratory endurance
7: cardiorespiratory test
8: aerobic fitness
9: 5 OR 6 OR 7 OR 8
10: adolescent
11: youth
12: teen
13: teenager
14: 10 OR 11 OR 12 OR 13
15: 4 AND 9 AND 14

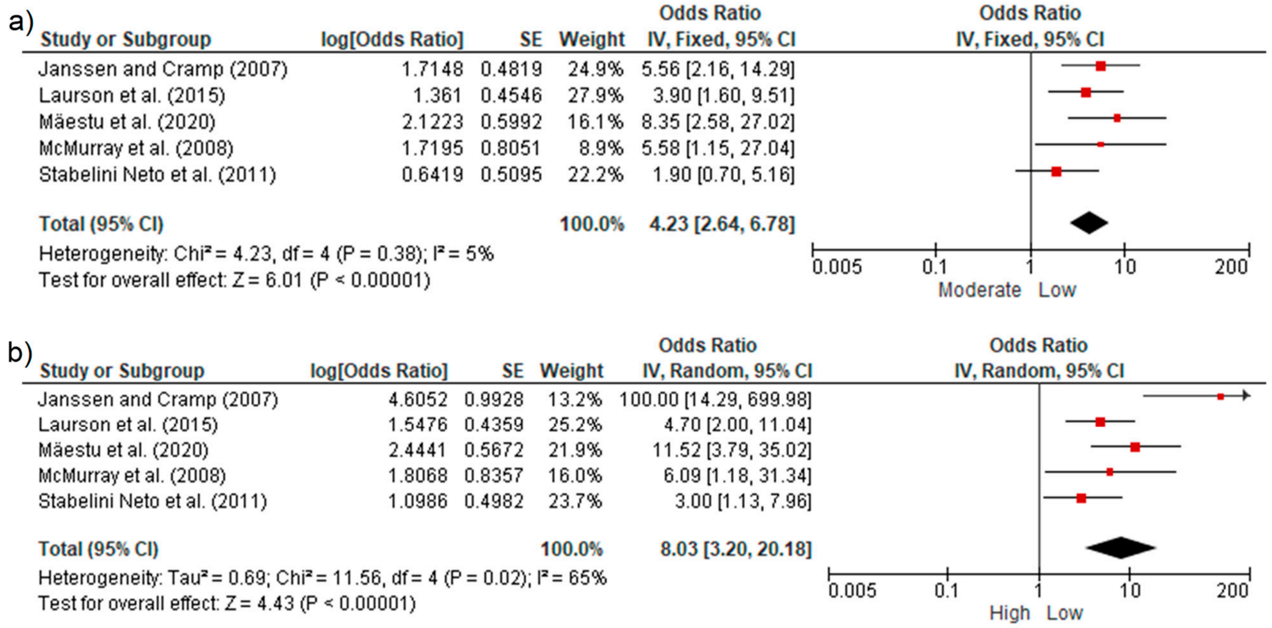

Supplementary Figure 1. Forest plot of subgroup analysis demonstrating the odds ratio for metabolic syndrome among adolescents with: a) moderate vs low cardiorespiratory fitness; b) high vs low cardiorespiratory fitness.

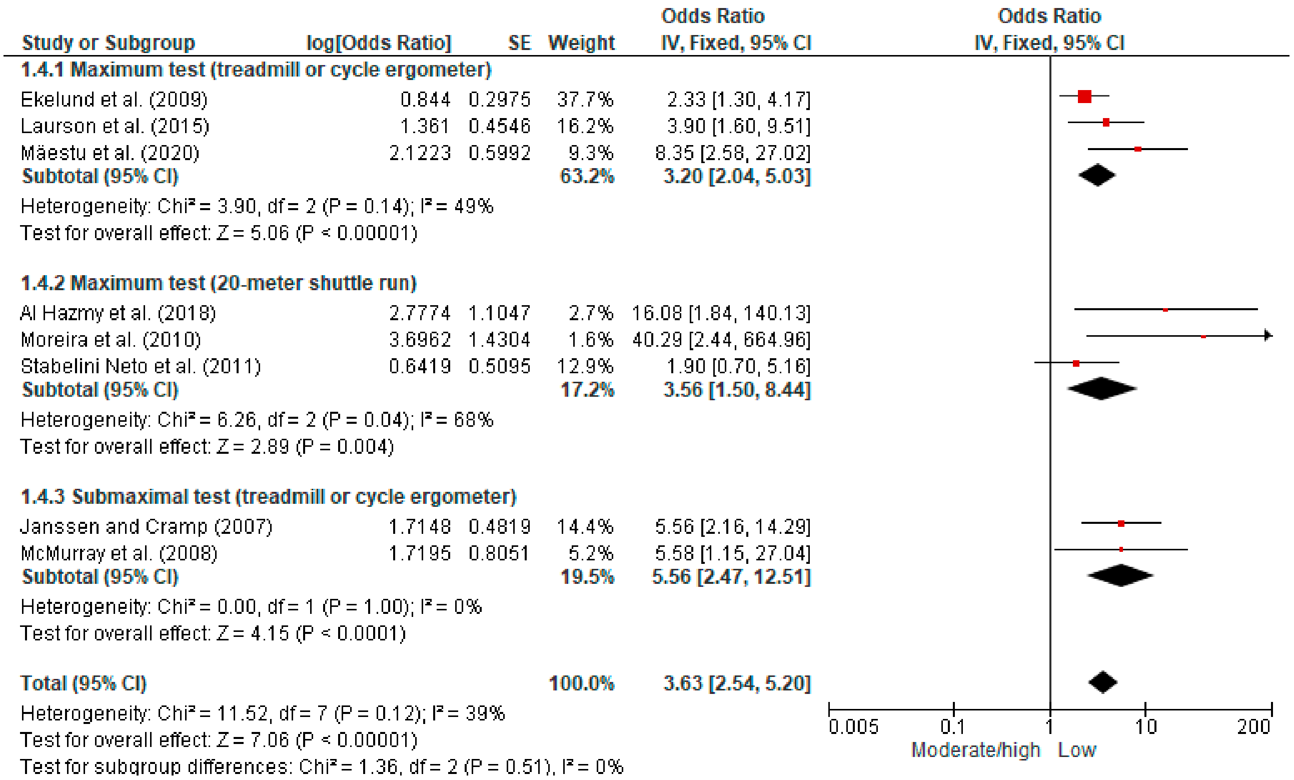

Supplementary Figure 2. Forest plot of the subgroup analysis demonstrating the odds ratio for metabolic syndrome among adolescents with moderate/high vs low cardiorespiratory fitness according to the type of test applied.
